# Clinical values of resting electrocardiography in patients with known or suspected chronic coronary artery disease: a stress perfusion cardiac MRI study

**DOI:** 10.1186/s12872-021-02440-5

**Published:** 2021-12-28

**Authors:** Yodying Kaolawanich, Rawiwan Thongsongsang, Thammarak Songsangjinda, Thananya Boonyasirinant

**Affiliations:** 1 Division of Cardiology, Department of Medicine, Faculty of Medicine Siriraj Hospital, Mahidol University, 2 Wanglang Road, Bangkok Noi, Bangkok, 10700 Thailand; 2 Division of Cardiology, Department of Medicine, Nakhon Pathom Hospital, Nakhon Pathom, Thailand

**Keywords:** Coronary artery disease, Electrocardiography, Myocardial ischemia, Cardiac magnetic resonance

## Abstract

**Background:**

Electrocardiography (ECG) is an essential investigation in patients with chronic coronary artery disease (CAD). However, evidence regarding the diagnostic and prognostic value of ECG in this population is limited. Therefore, we sought to determine whether baseline ECG abnormalities were associated with myocardial ischemia and cardiac events in patients with known or suspected chronic CAD.

**Methods:**

Consecutive patients with known (n = 146) or suspected chronic CAD (n = 349) referred for adenosine stress cardiac magnetic resonance (CMR) between 2011 and 2014 were enrolled. Resting ECGs were classified as major, minor, and no abnormalities. Predictors of myocardial ischemia on CMR and major adverse cardiac events (MACE) including cardiac death, nonfatal myocardial infarction, hospitalization for heart failure and late revascularization (> 180 days after CMR) were evaluated.

**Results:**

Average age was 69 ± 11 years (51% men). One hundred and eighty-five patients (37.4%) had major and 154 (31.1%) had minor ECG abnormalities. In patients with suspected CAD, myocardial ischemia was presented in 83 patients (23.8%). Multivariable analysis demonstrated major ECG abnormality as the strongest predictor of myocardial ischemia (HR 2.51; 95% CI 1.44–4.36; *p* = 0.001). Adding ECG to clinical pretest probability models improved the prediction of myocardial ischemia in ROC analyses (*p* = 0.04). In the whole cohort (n = 495), 91 MACE occurred during the median follow-up period of 4.8 years. Multivariable analysis showed that diabetes mellites, history of heart failure, prior revascularization, left ventricular ejection fraction, ischemia, and major ECG abnormality were independent predictors of MACE.

**Conclusion:**

Abnormal resting ECG is common in patients with known or suspected chronic CAD. ECG had important diagnostic and prognostic values in this population.

**Supplementary Information:**

The online version contains supplementary material available at 10.1186/s12872-021-02440-5.

## Introduction

Coronary artery disease (CAD) is one of the leading causes of morbidity and mortality worldwide [1]. Assessment of the pretest probability of CAD is crucial to select the most appropriate diagnostic test. The Diamond-Forrester (DF) model is one of the most common models used for this purpose, as recommended by the current guideline [2]. However, recent studies have demonstrated that the DF score was not adequate for modern populations of patients investigated for CAD [3–6]. The newly revised scores of the CAD consortium model may provide a more precise estimation of obstructive CAD [3–6]. Nevertheless, although the use of various pretest probability models as well as other noninvasive investigations have grown substantially, a landmark study demonstrated that only slightly more than one-third of patients without known disease who underwent elective cardiac catheterization had obstructive CAD [7].

Electrocardiography (ECG) is a fundamental investigation in CAD patients, especially for acute coronary syndrome (ACS). ECG not only helps to establish the diagnosis of acute myocardial infarction (MI) but also provides valuable information on infarct location, results of reperfusion, as well as prognosis [8, 9]. For chronic CAD, the current guideline recommended resting 12-lead ECG as an initial investigation in all patients with suspected CAD [2]. However, limited evidence exists regarding specific findings on resting ECG to provide a diagnostic clue in this population. Moreover, although ECG is a cheap and convenient investigation, information on resting ECG has never been included in any pretest probability model.

Resting ECG abnormality has been shown to be a strong predictor for mortality and major adverse cardiac events (MACE) in healthy subjects as well as for high-risk populations [10, 11]. We hypothesized that resting ECG abnormality may have a diagnostic and prognostic role in patients with known or suspected CAD.

The primary objective of this study was to determine whether abnormal resting ECG was a predictor of myocardial ischemia using adenosine stress cardiac magnetic resonance (CMR). The secondary objective was to evaluate the prognostic value of ECG in patients with known or suspected chronic CAD.

## Methods

### Study population

**Fig. 1:**
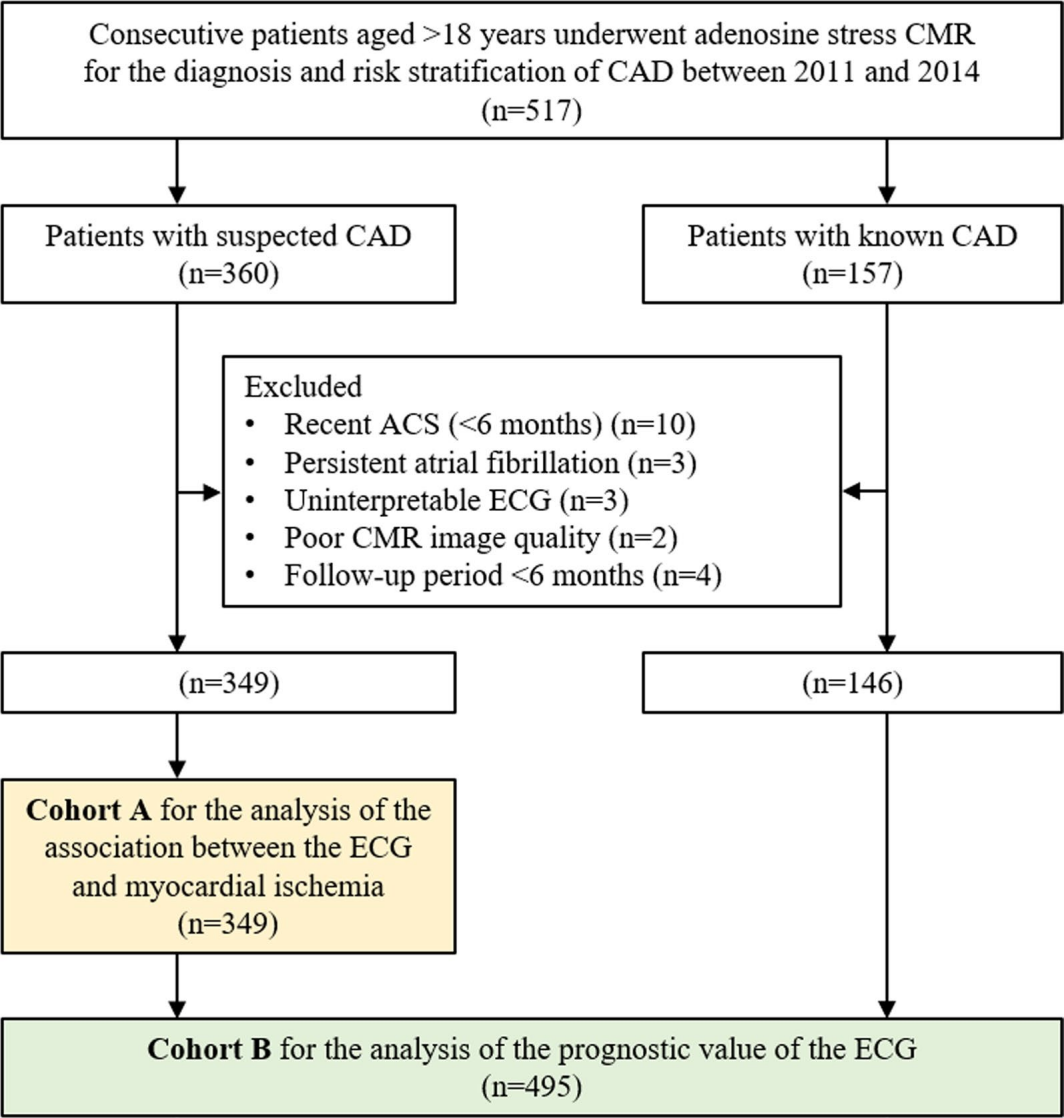
Study flow chart. ACS = acute coronary syndrome; CAD = coronary artery disease; CMR = cardiac magnetic resonance; ECG = electrocardiography

Consecutive patients over 18 years old with suspected or known CAD referred for adenosine stress CMR between May 2011 and December 2014 were included (Fig. 1). Known CAD (prior CMR) was defined using (i) history of MI, (ii) abnormal stress test, (iii) presence of significant CAD on coronary angiography (> 70% stenosis of three vessels or > 50% stenosis of the left main coronary artery), and (iv) history of coronary revascularization including percutaneous intervention or coronary artery bypass graphing. Patients with a diagnosis of recent ACS (< 6 months) were excluded due to dynamic change of ECG in this population. Patients with persistent atrial fibrillation, unreadable ECG, poor CMR image quality, or follow-up time less than 6 months were also excluded. After exclusion, patients were divided into 1) cohort A including patients with suspected CAD only (no known CAD) and 2) cohort B including patients with suspected CAD (cohort A) plus known CAD. Patients in cohort A were analyzed to determine whether the resting ECG was a predictor of myocardial ischemia, while patients in cohort B were analyzed to assess the prognostic role of ECG (Fig. 1). The study was done in accordance with the Declaration of Helsinki. The institutional ethics committee (Siriraj Institutional Review Board [SIRB], Faculty of Medicine Siriraj Hospital, Mahidol University) approved this retrospective study and waived the need for additional written informed consent.

### ECG data

Twelve-lead ECG was obtained on the CMR date as a routine protocol for adenosine stress CMR. Each ECG was reviewed by two trained cardiologists and discordant results were adjudicated by a senior cardiologist.

ECGs were coded according to the Minnesota Code (MC) and categorized into three groups as major, minor and no abnormalities based on the MC and according to previous publications [10–13]. Criteria for major ECG abnormalities were any of the following: Q-QS wave abnormalities (MC 1–1 to 1–2–8); left ventricular hypertrophy (LVH) (MC 3–1); complete bundle branch block or intraventricular block (MC 7–1–1, 7–2–1, 7–4 or 7–8); atrial fibrillation (AF) or atrial flutter (MC 8–3); or major ST-T changes (MC 4–1, 4–2, 5–1 and 5–2). Criteria for minor ECG abnormalities were minor ST-T changes (MC 4–3, 4–4, 5–3 and 5–4) or minor/isolated Q waves defined as MC 1–3 that are not codable as MI in the absence of any other major Q waves [10, 12]. Patients with both major and minor abnormalities were classified as having major abnormalities. Patients without minor or major ECG abnormalities were classified as having no ECG abnormality.

### *CMR protocol* [14, 15]

A CMR study was performed to assess cardiac function, myocardial perfusion, and late gadolinium enhancement (LGE) using a 1.5 Tesla Philips Achieva XR scanner (Philips Medical Systems, Best, The Netherlands).

The cardiac functional study was performed by acquiring the images using a steady-state free precession (SSFP) technique in a vertical long axis, 2-chamber, 4-chamber, and multiple slice short-axis views. Parameters for cardiac function were echo time (TE) 1.8 ms (ms), repetitive time (TR) 3.7 ms, number of excitations 2, field of view (FOV) 390 × 312 mm, matrix 256 × 240, reconstruction pixels 1.52 × 1.21, slide thickness 8 mm and flip angle 70 degrees.

The myocardial first-pass perfusion study was performed by injection of 0.05 mmol/kg of gadolinium contrast agent (Magnevist, Bayer Schering Pharma, Berlin, Germany) at a rate of 4 ml/s immediately after a 4-min infusion of 140 mcg/kg/min of adenosine. Three short-axis slices of basal, mid and apical left ventricular (LV) levels were acquired using an ECG-triggered SSFP, inversion-recovery, single-shot, turbo gradient-echo sequence. Image parameters were TE 1.32 ms, TR 2.6 ms, flip angle 50 degrees, slice thickness 8 mm, FOV 270 mm and reconstructed FOV 320 mm.

LGE images were acquired approximately 10 min after an additional bolus of gadolinium (0.1 mmol/kg, rate 4 ml/s) by the 3D segmented-gradient-echo inversion-recovery sequence. LGE images were acquired in multiple short-axis slices at levels similar to the functional images, long axis, 2-chamber and 4-chamber view. Parameters for LGE study were TE 1.25 ms, TR 4.1 ms, flip angle 15 degrees, FOV 303 × 384 mm, matrix 240 × 256, in-plane resolution 1.26 × 1.5 mm, slice thickness 8 mm and 1.5 sensitivity-encoding factor.

### *Image analysis* [15, 16]

Standard LV volumes, mass, and ejection fraction (EF) were quantitatively measured from the stack of short-axis SSFP cine images.

The perfusion and LGE images were analyzed using visual assessment and consensus by two CMR-trained physicians blinded to clinical and follow-up data. Segmentation of each slice was performed according to the recommendation of the American Heart Association [17]. Perfusion images were read, and each of the 16 segments was visualized (segment-17 at the apex was not visualized). Inducible ischemia was defined as a subendocardial perfusion defect that (i) persisted beyond peak myocardial enhancement and for several RR intervals, (ii) was more than two pixels wide, (iii) followed one or more coronary arteries, and (iv) showed absence of LGE in the same segment [16]. Dark-banding artifacts were recorded if an endocardial dark band appeared at the arrival of contrast in the LV cavity before contrast arrival in the myocardium [16]. LGE images were also analyzed using visual assessment. LGE was considered present only if confirmed on both the short-axis and at least one other orthogonal plane [16].

### Clinical follow-up

Follow-up data were collected from clinical visits and medical records. Clinical event adjudication was completely blinded to clinical and CMR data. Patients were followed up for MACE. MACE was defined as the composite outcomes of cardiac mortality, nonfatal MI, hospitalization for unstable angina, hospitalization for heart failure, and late coronary revascularization (> 180 days after CMR). Need for revascularization therapy within 180 days after the CMR was considered to be triggered by the CMR results and therefore censored from MACE. Cardiac mortality included death resulting from acute MI, heart failure, sudden cardiac death, or death due to complications in cardiac procedures [18].

### Statistical analysis

Statistical analyses were performed using IBM SPSS Statistics for Windows version 20.0 (IBM Corp., Armonk, NY, USA). Continuous variables with normal distribution were presented as mean ± standard deviation (SD), and continuous variables with non-normal distribution were presented as median and interquartile ranges. The normality of the distribution of variables was examined by the Kolmogorov–Smirnov test. Categorical variables were present as absolute numbers and percentages. Clinical characteristics including CMR findings were compared among patients with major, minor, and no ECG abnormalities as well as patients with and without MACE. Normally distributed continuous data of multiple (> 2) groups were compared using one-way analysis of variance. Non-normally distributed continuous data of multiple (> 2) groups were compared using the Kruskal–Wallis test. Multiple comparisons were analyzed using the Scheffé's method.

Continuous variables between two groups were compared using the student’s unpaired t-test or Mann–Whitney U test. Categorical data were compared by the chi-squared test or Fisher’s exact test, as appropriate.

To analyze the predictors of myocardial ischemia, a Cox-regression analysis was performed to assess univariable predictors from baseline characteristics and ECG. We included conventional risk factors such as age, sex, diabetes mellitus, and the variables which showed significance by univariable analysis (*p* < 0.05) to enter the multivariable analysis. The multivariable analysis was divided to 2 models according to the pretest probability models to predict obstructive CAD (model 1 by DF model and model 2 by clinical model of CAD consortium.

The incremental value of ECG in predicting myocardial ischemia was assessed by comparing the area under the receiver operating characteristic (ROC) curves for the DF model and clinical model of CAD consortium with and without the addition of ECG. The ROC curves were compare using C-statistic method.

Kaplan–Meier plots were used to compare the proportion of patients in each group who had an event during follow-up. The log-rank test was used to compare groups on the Kaplan–Meier analysis. To analyze the predictors of MACE, a Cox-regression analysis was performed to assess univariable predictors from baseline characteristics and CMR parameters. We included conventional risk factors such as age, sex, diabetes mellitus, and the variables which showed significance by univariable analysis (*p* < 0.05) to enter the multivariable analysis. To assess the incremental prognosis values of multiple major ECG abnormalities, global chi-square values were calculated after adding predictors in the following order: clinical, numbers of major ECG abnormalities (1, 2, and more than 2 findings).

The hazard ratios (HRs) and 95% confidence intervals (CIs) of the outcomes were calculated, with a *p *value < 0.05 considered statistically significant.

## Results

### Patient characteristics

Among the 517 patients with known or suspected CAD that were included (Fig. 1), 22 were excluded: 10 had a history of recent ACS, 3 had persistent AF, 3 had unreadable ECGs, 2 had poor CMR image quality, and 4 had a follow-up period of less than 6 months. Thus, 349 patients with suspected CAD (Cohort A) were included in the final analysis of the association between the ECG and myocardial ischemia. After adding 146 patients with known CAD, a total of 495 patients (Cohort B) were included in the final analysis of the prognostic value of the ECG (Fig. 1).

Clinical characteristics of patients with suspected CAD (Cohort A, n = 349) are listed in Table 1. The average age was 68.3 years, 46.7% were men. The most common presenting symptom was atypical angina (30.4%). The pretest probability of obstructive CAD was intermediate (20.3% by the DF model, and 30.3% by clinical model of CAD consortium). The average LVEF was 68.8%. Myocardial ischemia was presented in 83 patients (23.8%) and LGE was detected in 59 patients (16.9%). No microvascular obstruction (representing recent MI) was detected by LGE-CMR.

**Table 1:** Clinical Characteristics of Patients with Suspected CAD (Cohort A)

	All	Major ECG abnormality^a^	Minor ECG abnormality^a^	No ECG abnormality	*P *value
	(n = 349)	(n = 106)	(n = 121)	(n = 122)	
Age, years	68.3 ± 10.9	67.5 ± 11.2	68.0 ± 11.0	69.4 ± 10.7	0.41
Men	163 (46.7)	51 (48.1)	47 (38.8)	65 (53.3)	0.07
Body mass index kg/m^2^	26.9 ± 4.4	26.3 ± 4.2	27.7 ± 4.7	26.7 ± 4.3	**0.04**^**†**^
Systolic blood pressure, mmHg	136.2 ± 19.4	134.1 ± 19.4	137.7 ± 21.1	136.62 ± 17.5	0.38
Diastolic blood pressure, mmHg	73.5 ± 12.1	73.2 ± 12.1	74.3 ± 12.7	73.0 ± 11.7	0.69
Heart rate, beats/minute	77.9 ± 13.7	77.9 ± 15.2	77.9 ± 13.6	77.8 ± 12.5	0.99
Typical angina	40 (11.5)	14 (13.2)	11 (9.1)	15 (12.3)	0.59
Atypical angina	106 (30.4)	27 (25.5)	39 (32.2)	40 (32.8)	0.42
Dyspnea	53 (15.2)	17 (16.0)	17 (14.1)	19 (15.6)	0.91
History of heart failure	34 (9.7)	16 (15.1)	10 (8.3)	8 (6.6)	0.08
*CAD risk factors*					
Hypertension	317 (90.8)	95 (89.6)	109 (90.1)	113 (92.6)	0.7
Hyperlipidemia	275 (78.8)	81 (76.4)	99 (81.8)	95 (77.9)	0.58
Diabetes mellitus	202 (57.9)	64 (60.4)	72 (59.5)	66 (54.1)	0.57
Cigarette smoking	61 (17.5)	29 (27.4)	14 (11.6)	18 (14.8)	0.01^*†^
*Pretest probability of obstructive CAD, %*					
Diamond-forrester	20.3 ± 10.9	19.7 ± 10.7	18.6 ± 10.5	22.6 ± 11.2	0.01^*‡^
CAD consortium, clinical model	30.3 ± 20.4	29.7 ± 19.3	28.2 ± 20.9	32.8 ± 20.9	0.2
*Medications*					
ACEI or ARB	150 (43.0)	49 (46.2)	51 (42.2)	50 (41.0)	0.71
Antiplatelet	152 (43.6)	49 (46.2)	50 (41.3)	53 (43.4)	0.76
Beta blocker	155 (44.4)	48 (45.3)	46 (38.0)	61 (50.0)	0.17
Calcium channel blocker	120 (34.4)	30 (28.3)	41 (33.9)	49 (40.2)	0.17
Statin	174 (49.9)	50 (47.2)	61 (50.4)	63 (51.6)	0.79
*CMR*					
LV end diastolic volume index, ml/m^2^	74.5 ± 24.7	84.3 ± 35.0	70.6 ± 18.1	70.0 ± 15.5	< 0.001^*†^
LV end systolic volume index, ml/m^2^	25.8 ± 23.3	35.1 ± 34.6	23.7 ± 17.6	19.6 ± 9.9	< 0.001^*†‡^
LV ejection fraction, %	68.8 ± 13.3	64.1 ± 16.8	68.7 ± 12.3	72.9 ± 8.9	< 0.001^*†^
LV mass index, g/m^2^	51.6 ± 17.6	60.8 ± 23.1	47.0 ± 11.7	48.2 ± 13.4	< 0.001^*†‡^
Myocardial ischemia	83 (23.8)	39 (36.8)	23 (19.0)	21 (17.2)	0.001^*†^
Late gadolinium enhancement	59 (16.9)	39 (36.8)	13 (10.7)	7 (5.7)	< 0.001^*†^

Values are numbers (percentages) or mean ± standard deviation. Bold values are < 0.05

ACEI = angiotensin-converting enzyme inhibitor; ARB = angiotensin II receptor blocker; CAD = coronary artery disease; CMR = cardiac magnetic resonance; ECG = electrocardiographic; LV = left ventricular

*Major ECG abnormality versus no ECG abnormality

^†^major ECG abnormality versus minor ECG abnormality; ^‡^ minor ECG abnormality versus no ECG abnormality

^a^See “Methods” section for definitions of major and minor ECG abnormalities

All ECGs were performed on the same day as CMR. No patient reported ongoing chest pain during ECG. Major ECG abnormality was detected in 106 patients (30.4%) including Q-Qs wave (n = 34), LV hypertrophy (n = 25), complete bundle branch block/intraventricular block (n = 26), atrial fibrillation (n = 6) and major ST-T change (n = 36). Minor ECG abnormality was detected in 121 patients (34.6%) including minor ST-T change (n = 68) and minor/isolated Q wave (n = 71). Among a random sample of 10% of ECGs, Kappa values for the categorization described were 0.85 for major, 0.80 for minor and 0.81 for no ECG abnormalities.

There was no significant difference in the pretest probability of obstructive CAD using the clinical model of CAD consortium (*p* = 0.20) among the three groups. However, using the DF model, patients with no ECG abnormality had a higher pretest probability of obstructive CAD than those with major or minor abnormality (*p* = 0.01). Patients with major ECG abnormality had a higher prevalence of cigarette smoking compared with minor or no abnormality. Patients with major ECG abnormality also had significantly higher LV volume, higher LV mass index and lower LVEF than minor and no ECG abnormality (*p* < 0.001 for all). Moreover, patients with major ECG abnormality demonstrated a significantly higher prevalence of myocardial ischemia and LGE (*p* < 0.001 and *p* = 0.001, respectively).

### Predictors of myocardial ischemia

Table 2 demonstrates the univariable and multivariable analyses of the predictors of myocardial ischemia. Men, diabetes mellitus, cigarette smoking, both models of pretest probability of obstructive CAD, taking antiplatelets and major ECG abnormality were predictors of myocardial ischemia in the univariable analysis. For both models of the multivariable analysis (model 1 using the DF and model 2 using the clinical model of CAD consortium), major ECG abnormality was the strongest predictor of myocardial ischemia (*p* = 0.001) Minor ECG abnormality was not associated with myocardial ischemia.

**Table 2:** Predictors of Myocardial Ischemia

	Univariable analysis		Multivariable analysis			
			Model 1 (diamond-forrester)		Model 2 (CAD consortium, clinical model)	
	Odd ratio (95%CI)	*P* value	Odd ratio (95%CI)	*P* value	Odd ratio (95%CI)	*P* value
Age	1.01 (0.99, 1.04)	0.27	0.99 (0.96, 1.02)	0.6	0.99 (0.95, 1.02)	0.38
Men	1.69 (1.03, 2.78)	**0.04**	0.47 (0.18, 1.19)	0.11	0.71 (0.35, 1.44)	0.34
Body mass index	0.95 (0.89, 1.003)	0.06				
Systolic blood pressure	1.02 (0.99, 1.03)	0.07				
Diastolic blood pressure	0.99 (0.97, 1.01)	0.22				
Heart rate	0.99 (0.98, 1.01)	0.54				
Hypertension	2.32 (0.79, 6.83)	0.13				
Hyperlipidemia	1.44 (0.76, 2.73)	0.27				
Diabetes mellitus	1.71 (1.02, 2.86)	**0.04**	1.53 (0.86, 2.72)	0.15	1.03 (0.54, 1.98)	0.92
Cigarette smoking	2.52 (1.40, 4.53)	**0.002**	2.26 (1.15, 4.43)	**0.02**	1.70 (0.86, 3.39)	0.13
History of heart failure	1.38 (0.63, 3.02)	0.42	1.60 (0.66, 3.89)	0.3	1.50 (0.62, 3.63)	0.36
*Pretest probability of obstructive CAD*						
Diamond-forrester	1.04 (1.02, 1.07)	** < 0.001**	1.08 (1.03, 1.12)	**0.002**	–	–
CAD consortium, clinical model	1.03 (1.02, 1.04)	** < 0.001**	–	–	1.04 (1.02, 1.06)	**0.001**
*Medications*						
ACEI or ARB	0.90 (0.54, 1.48)	0.67				
Antiplatelet	1.76 (1.07, 2.89)	**0.03**	1.71 (1.00, 2.91)	0.05	1.68 (1.00, 2.87)	0.05
Beta blocker	0.95 (0.58, 1.56)	0.83				
Calcium channel blocker	0.84 (0.49, 1.42)	0.5				
Statin	1.04 (0.64, 1.70)	0.88				
*ECG*						
No ECG abnormality (reference)	1	–				
Major ECG abnormality^a^	2.80 (1.52, 5.17)	** < 0.001**	2.51 (1.44, 4.36)	**0.001**	2.64 (1.51, 4.62)	**0.001**
Minor ECG abnormality^a^	1.13 (0.59, 2.17)	0.72				

Bold values are < 0.05

CI = confidence interval; other abbreviations as in Table 1

^a^See “Methods” section for definitions of major and minor ECG abnormalities

To determine whether any ECG findings were more specific for myocardial ischemia, we performed a stratified analysis by types of ECG. Additional file 1: Table 1 shows an exploratory analysis of specific ECG findings and the risk of myocardial ischemia. Highest risk of myocardial ischemia was observed in patients with Q-Qs wave (47%; 95% CI 29% to 65%) and major ST-T changes (47%; 95% CI 30 to 64%). Both types of minor ECG abnormality (minor ST-T change and minor/isolated Q wave) showed a similar risk of myocardial ischemia compared with no ECG abnormality (range 17 to 19%).

**Fig. 2:**
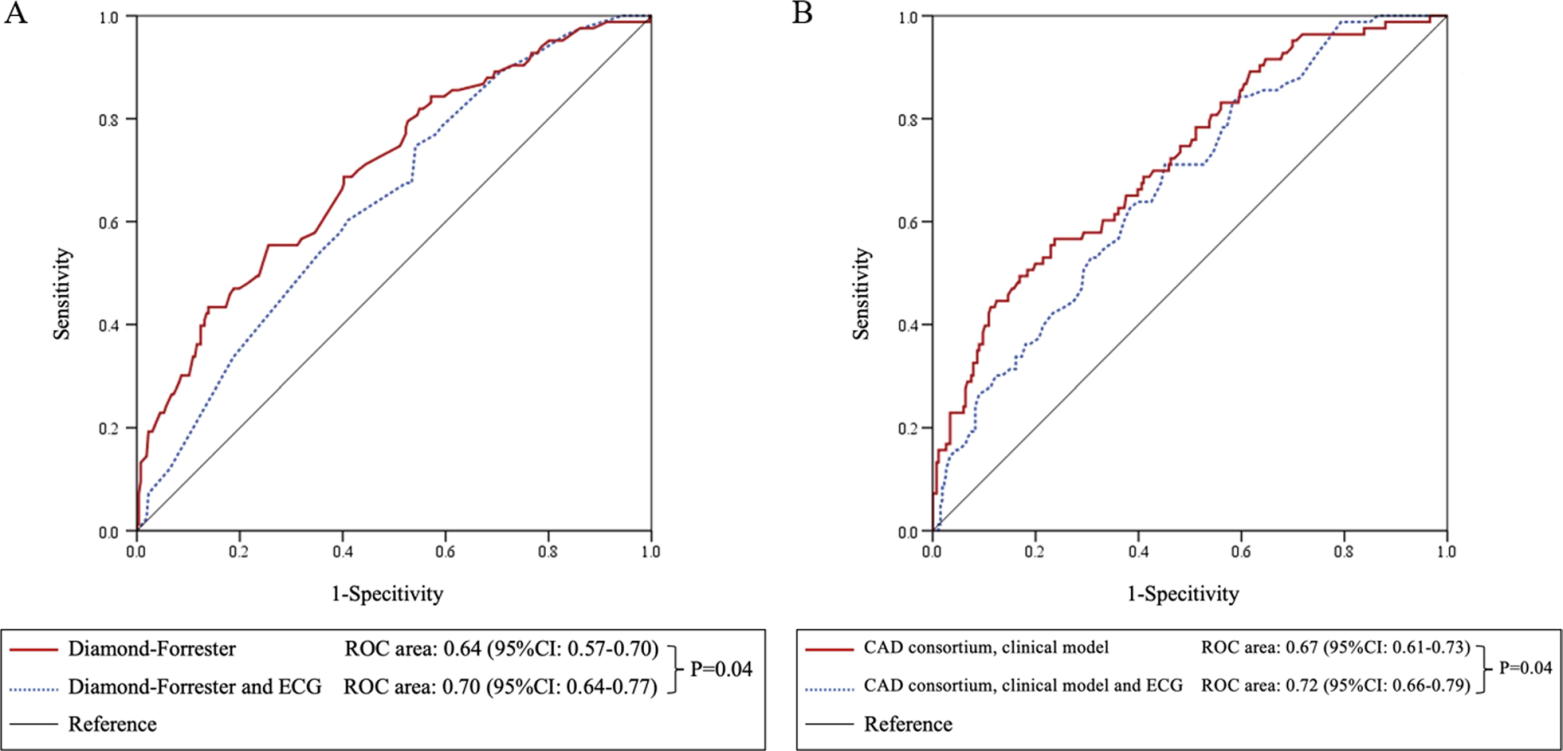
Receiver-operating characteristics (ROC) curves for prediction of myocardial ischemia on adenosine stress perfusion CMR. **A** ROC curves derived from the Diamond-Forester (DF) model (dotted blue line) and the DF model plus ECG (red line). **B** ROC curves derived from the clinical model of CAD consortium (dotted blue line) and the clinical model of CAD consortium plus ECG (red line)

We also performed additional analyses using two models of pretest probability of obstructive CAD in predicting myocardial ischemia with and without ECG data to explore how adding ECG data might improve the risk prediction for myocardial ischemia. The area under the curve for predicting myocardial ischemia using the DF score was 0.64, which increased to 0.70 when adding ECG data (*p* = 0.04) (Fig. 2a). Similar to the clinical model of CAD consortium, after adding ECG data, the area under the curve increased from 0.67 to 0.72 (*p* = 0.04) (Fig. 2b).

### Follow-up results

Cohort B was composed of 349 patients with suspected and 146 patients with known CAD. During a median follow-up of 4.8 years (2.9, 5.6 years), 91 MACE (18.4%) occurred. The rate of specific cardiac events is listed in Additional file 1: Table 2. Seventy-eight revascularization procedures were performed less than 180 days after CMR with a median time of 40 days. Most of the indications were significant myocardial ischemia on stress CMR images. These results were censored from MACE.

**Fig. 3:**
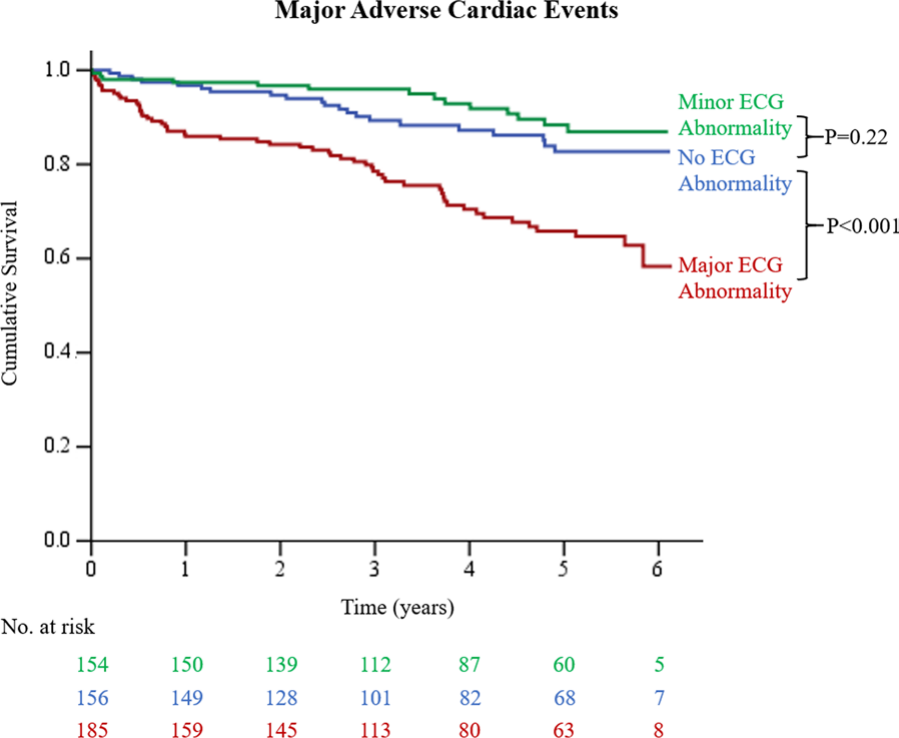
Kaplan–Meier survival curves for major adverse cardiac events (MACE) stratified by three types of ECG. Kaplan–Meier curves demonstrate a significantly higher rate of MACE in patients with major ECG abnormality (*p* < 0.001)

Table 3 shows the clinical characteristics of patients with and without MACE. Patients with MACE were more likely to have a history of CAD, MI, heart failure, coronary revascularization, major ECG abnormality as well as lower LVEF, myocardial ischemia, and LGE. Figure 3 demonstrates the Kaplan–Meier estimates of MACE for patients without ECG abnormalities versus major and minor ECG abnormalities. Patients with major ECG abnormalities had a significantly higher rate of MACE compared to those with no ECG abnormality (HR 2.48; 95% CI 1.50–4.09; *p* < 0.001). Patients with minor ECG abnormalities had similar rates of MACE compared with those with no ECG abnormality (HR 0.65; 95% CI 0.33–1.28; *p* = 0.22). Multivariable analysis showed diabetes mellitus, prior revascularization, history of heart failure, major ECG abnormality, LVEF, and myocardial ischemia as independent predictors of MACE (Table 4).

**Table 3:** Clinical Characteristics of Patients with and without MACE (Cohort B)

	All (n = 495)	MACE (n = 91)	No MACE (n = 404)	*P *value
Age, years	68.9 ± 10.6	70.5 ± 10.5	68.5 ± 10.7	0.1
Men	253 (51.1)	49 (53.9)	204 (50.5)	0.56
Body mass index, kg/m^2^	26.6 ± 4.4	25.9 ± 4.2	26.8 ± 4.4	0.08
Systolic blood pressure, mmHg	137.0 ± 19.9	137.4 ± 22.8	136.9 ± 19.1	0.84
Diastolic blood pressure, mmHg	73.0 ± 12.0	69.9 ± 11.7	73.7 ± 12.0	**0.01**
Heart rate, beats/minute	77.1 ± 13.7	76.8 ± 14.4	77.1 ± 13.6	0.83
*CAD risk factors*				
Hypertension	449 (90.7)	86 (94.5)	363 (89.9)	0.17
Hyperlipidemia	406 (82.0)	81 (89.0)	325 (80.5)	0.06
Diabetes mellitus	283 (57.2)	59 (64.8)	224 (55.4)	0.1
Cigarette smoking	91 (18.4)	23 (25.3)	68 (16.8)	0.06
*Clinical history*				
History of CAD	143 (28.9)	52 (57.1)	91 (22.5)	** < 0.001**
History of myocardial infarction	25 (5.1)	9 (9.9)	16 (4.0)	**0.03**
Prior revascularization	99 (20.0)	36 (39.6)	63 (15.6)	** < 0.001**
History of heart failure	55 (11.1)	25 (27.5)	30 (7.4)	** < 0.001**
*Medications*				
ACEI or ARB	231 (46.7)	51 (56.0)	180 (44.6)	**0.04**
Antiplatelet	267 (53.9)	59 (64.8)	208 (51.5)	**0.02**
Beta blocker	250 (50.5)	50 (55.0)	200 (49.5)	0.35
Calcium channel blocker	160 (32.3)	27 (29.7)	133 (32.9)	0.55
Statin	277 (56.0)	59 (64.8)	218 (54.0)	0.06
ECG^a^				
QRS axis, degree	25.4 ± 37.7	22.2 ± 35.7	26.1 ± 38.1	0.46
No ECG abnormality	156 (31.5)	21 (23.1)	135 (33.4)	0.06
Major ECG abnormality	185 (37.4)	56 (61.5)	129 (31.9)	** < 0.001**
Q-Qs wave	76 (15.4)	25 (27.5)	51 (12.6)	** < 0.001**
LV hypertrophy	43 (8.7)	17 (18.7)	26 (6.4)	** < 0.001**
Complete bundle branch/IVB	38 (7.7)	6 (6.6)	32 (7.9)	0.67
Atrial fibrillation/flutter	12 (2.4)	6 (6.6)	6 (1.5)	**0.01**
Major ST-T changes	64 (12.9)	25 (27.5)	39 (9.7)	** < 0.001**
Minor ECG abnormality	154 (31.1)	14 (15.4)	140 (34.7)	** < 0.001**
Minor ST-T changes	95 (19.2)	11 (12.1)	84 (20.8)	0.06
Minor/isolated Q wave	86 (17.4)	6 (6.6)	80 (19.8)	**0.003**
*CMR*				
LV end diastolic volume index, ml/m^2^	77.6 ± 26.7	91.9 ± 37.6	74.4 ± 22.5	** < 0.001**
LV end systolic volume index, ml/m [2]	29.3 ± 26.6	45.0 ± 38.3	25.7 ± 21.7	** < 0.001**
LV ejection fraction, %	66.5 ± 15.0	58.2 ± 19.9	68.4 ± 13.0	** < 0.001**
LV mass index, g/m^2^	53.1 ± 17.4	62.0 ± 21.0	51.0 ± 15.8	** < 0.001**
Myocardial ischemia	153 (30.9)	49 (53.9)	104 (25.7)	** < 0.001**
Late gadolinium enhancement	146 (29.4)	51 (56.0)	95 (23.5)	** < 0.001**

Values are numbers (percentages) or mean ± standard deviation. Bold values are < 0.05

IVB = intraventricular block; MACE = major adverse cardiac events; other abbreviations as in Table 1

^a^See “Methods” section for definitions of major and minor ECG abnormalities

**Table 4:** Predictors of MACE

	Univariable analysis		Multivariable analysis	
	HR (95%CI)	*P* value	HR (95%CI)	*P* value
Age	1.02 (0.99, 1.04)	0.13	1.01 (0.99, 1.03)	0.23
Men	1.06 (0.54, 2.05)	0.87	0.94 (0.60, 1.49)	0.8
Body mass index	0.95 (0.91, 1.001)	0.05		
Systolic blood pressure	0.99 (0.98, 1.01)	0.88		
Diastolic blood pressure	0.97 (0.96, 0.99)	**0.003**	0.98 (0.96, 1.00)	0.05
Heart rate	0.99 (0.98, 1.01)	0.92		
Hypertension	0.79 (0.28, 2.23)	0.65		
Hyperlipidemia	0.93 (0.39, 2.24)	0.87		
Diabetes mellitus	1.43 (0.73, 2.83)	0.3	1.77 (1.14, 2.76)	**0.01**
Cigarette smoking	1.05 (0.46, 2.40)	0.91		
History of CAD	3.56 (2.36, 5.40)	** < 0.001**	1.18 (0.59, 2.37)	0.64
History of myocardial infarction	2.55 (1.28, 5.08)	**0.01**	1.46 (0.67, 3.17)	0.35
Prior revascularization	3.09 (2.03, 4.71)	** < 0.001**	3.02 (1.94, 4.70)	** < 0.001**
History of heart failure	3.60 (2.27, 5.70)	** < 0.001**	2.70 (1.59, 4.58)	** < 0.001**
*Medications*				
ACEI or ARB	0.90 (0.46, 1.75)	0.75		
Antiplatelet	1.60 (0.80, 3.21)	0.19		
Beta blocker	0.96 (0.50, 1.87)	0.91		
Calcium channel blocker	1.20 (0.61, 2.39)	0.6		
Statin	1.85 (0.90, 3.77)	0.09		
*ECG*				
No ECG abnormality (reference)	1	-		
Major ECG abnormality^a^	2.48 (1.50, 4.09)	** < 0.001**	1.65 (1.02, 2.66)	**0.04**
Minor ECG abnormality^a^	0.65 (0.33, 1.28)	0.22		
*CMR*				
LV ejection fraction	0.97 (0.96, 0.98)	** < 0.001**	0.98 (0.97, 0.99)	**0.01**
LV mass index	1.02 (1.02, 1.03)	** < 0.001**	1.01 (1.00, 1.02)	0.25
Myocardial ischemia	3.03 (2.00, 4.57)	** < 0.001**	1.81 (1.17, 2.81)	**0.01**
Late gadolinium enhancement	3.78 (2.49, 5.72)	** < 0.001**	1.55 (0.89, 2.69)	0.12

Abbreviations as in Table 1 to 3

^a^See “Methods” section for definitions of major and minor ECG abnormalities

**Fig. 4:**
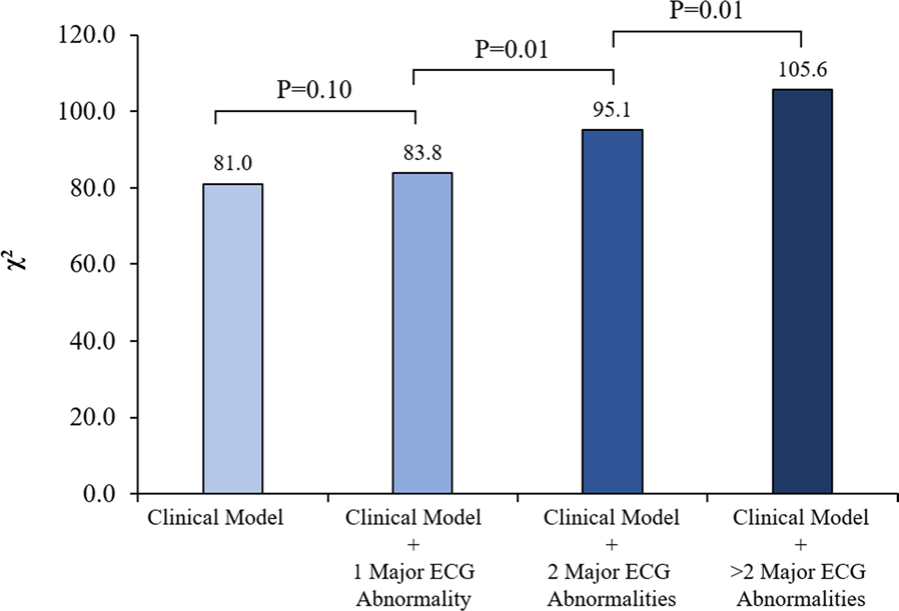
Incremental prognostic value of numbers of major ECG abnormalities for prediction of major adverse cardiac events (MACE). Clinical = age, male gender, diabetes mellitus, history of heart failure, and prior coronary revascularization.

Given that several patients may have more than one abnormality of ECG, we performed an additional analysis to determine whether numbers of major ECG abnormalities could provide an incremental prognostic value over the clinical model in hierarchical order (Fig. 4). The clinical model included age, male gender, diabetes mellitus, history of heart failure and prior revascularization. Multiple ECG abnormalities provided an incremental prognostic value over clinical data plus one ECG abnormality (*p* = 0.01).

## Discussion

Results of this study demonstrated that (i) the presence of major ECG abnormality was associated with a ≈threefold increased likelihood of myocardial ischemia in patients with an intermediate pretest probability of obstructive CAD, (ii) ECG provided an incremental diagnostic value over clinical pretest probability models, and (iii) ECG was an independent prognostic predictor for MACE in patients with known or suspected CAD.

Several previous studies reported an association between ECG abnormalities and cardiovascular outcomes [10, 11]. Auer et al. conducted a population-based study of 2,192 older adults. They found that the prevalence of baseline ECG abnormality was 36% and associated with significantly increased risks of coronary heart disease events [10]. Krittayaphong et al. demonstrated that ECG abnormalities including AF, Q-QS wave, intraventricular conduction delay and LVH increased the risk of MACE in patients with high cardiovascular risk [11]. However, these studies were conducted on patients without CAD. ECG is an essential investigation to make a diagnosis of STEMI, as well as providing important prognostic value [8, 9]. Nevertheless, evidence regarding the diagnostic and prognostic role of resting ECG in patients with chronic CAD is limited.

Farhan et al. demonstrated an association between T wave inversion in lead aVL and significant stenosis of mid LAD by invasive coronary angiography in patients with stable angina [19]. However, a relatively small number of populations precluded an accurate multivariate analysis to compare T wave inversion in lead aVL with other ECG findings [19]. Our data demonstrated that resting ECG abnormality was common in patients with known or suspected chronic CAD (37% for major and 31% for minor abnormality). The prevalence was similar to the study of Farhan et al. [19]. Our results demonstrated that major ECG abnormality was a strong and independent predictor of myocardial ischemia. Specifically, major ST-T change and Q-QS wave were the most predictive values.

In patients with chest pain, guidelines recommend initial diagnostic evaluation by assessment of an individual’s pretest probability of CAD to make decisions regarding further diagnostic testing [2]. Multiple risk scores have been developed to systematize risk assessment based on clinical history including the DF and CAD consortium models [5, 20]. However, recent studies demonstrated that the DF score overestimated the probability of CAD, especially in women [20]. The CAD consortium clinical model appeared to improve the prediction in low-risk patients but the use of this model requires caution in a high-risk population [6]. In our study, both the DF score and the CAD consortium clinical model overestimated myocardial ischemia using CMR as a reference. We used adenosine stress CMR to define obstructive CAD and this may cause some degree of discrepancies due to the referral bias of patients who underwent CMR. However, our data showed that ECG could add a predictive value over pretest probability score alone as a clinical implication of ECG in this population.

Minor ECG abnormality was common with prevalence of up to 40% in patients with abnormal signs or symptoms of CVD [21]. Daviglus et al. found that persistent nonspecific ST-T abnormality was associated with cardiovascular mortality in 1,673 healthy men [22]. In our study, the prevalence of minor ECG abnormality (minor ST-T change or minor/isolated Q wave) was 31%. However, in our study, minor ECG abnormalities were not associated with myocardial ischemia or MACE, due to differences in the definition of minor ECG abnormality and number of patients. A larger study may be required to confirm the results.

A number of studies demonstrated the prognostic value of ECG abnormality in various populations including healthy elderly and patients with hypertension and diabetes [10, 23, 24]. Abnormal ECG was found to be consistently associated with future cardiovascular events [10, 23, 24]. Our study showed a prognostic value of major ECG abnormality in patients with known or suspected CAD. The numbers of major ECG abnormalities also provided additional prognostic value over a single abnormality. Studies regarding the prognostic value of resting ECG in patients with chronic CAD are limited. However, our result was similar to Jeger et al. who demonstrated that resting ECG abnormalities including ST depression and faster heart rate were associated with cardiovascular events in patients with CAD who underwent major noncardiac surgery [25]. Q wave on resting ECG appears to represent previous MI. However, recent studies demonstrated that Q wave on an ECG may not be an accurate predictor for previous MI [26–28]. Our results supported this information. Given the very small number of patients with a history of MI (< 2%), LGE-CMR revealed the number of patients with unrecognized MI at approximately 15%. LGE was also an independent predictor for MACE, consistent with previous reports [26, 29].

Our results may be applied for the evaluation of patients with known or suspected CAD. ECG is a low-cost, non-invasive investigation. However, ECG cannot replace a conventional stress test but maybe a complementary tool to indicate the urgency of patient assessment, including a non-invasive stress test or coronary angiography. For example, patients with a moderate to high likelihood of obstructive CAD; major ECG abnormality may help the clinician decide whether the patients go directly for invasive coronary angiography.

Patients with CAD are at increased risk of adverse events. In addition to ECG abnormalities, anemia is also associated with an increased risk for mortality. Leonardi et al. demonstrated that among patients with ACS managed invasively, in-hospital hemoglobin drop ≥ 3 g/dl, even in the absence of overt bleeding, is common and is independently associated with increased risk for 1-year mortality [30]. Patients with CAD and high bleeding risk also were highlighted in the study of Corpataux et al. [31]. They validated the set of clinical and biochemical criteria proposed by consensus by the Academic Research Consortium (ARC) for High Bleeding Risk (HBR) to identify HBR patients undergoing percutaneous coronary intervention. Results showed that all major and the majority of minor ARC-HBR criteria identified in isolation patients at HBR [31]. Finally, antithrombotic therapy plays a central role in the secondary prevention of CAD. Oral P2Y_12_ inhibitors have mainly been investigated in combination with aspirin after coronary revascularization. However, dual antiplatelet therapy (DAPT) increases the risk of bleeding. A recent meta-analysis of Valgimigli et al. demonstrated that P2Y_12_ inhibitor monotherapy was associated with a similar risk of death and ischemic events and a lower bleeding risk compared with DAPT [32]. Aspirin cessation from one to three months after coronary revascularization and continuation with P2Y_12_ inhibitor monotherapy may be warranted instead of continuation of DAPT.

### Study limitations

This study had some limitations. Firstly, single-time ECG data may change dynamically during an ischemic event. However, no patient recorded chest pain during an ECG. Secondly, this study was conducted on patients with known or suspected CAD, referred for adenosine stress CMR. This may lead to a referral bias and the results may not be applicable for all patients with chronic CAD. Thirdly, we used adenosine perfusion CMR to define obstructive CAD and not all patients underwent invasive coronary angiography. However, adenosine stress CMR demonstrated very high accuracy compared with invasive fractional flow reserve [33]. Finally, we did not include revascularization procedures that occurred less than 180 days after CMR. Although most of the indications for revascularization were the presence of ischemia on CMR, interpretation of the results may have a limitation for this population.

## Conclusions

Abnormal ECG was common in patients with known or suspected chronic CAD. In a cohort of patients who had intermediate pretest probability of obstructive CAD, major ECG abnormality was associated with myocardial ischemia and provided an incremental predictive value over clinical pretest probability models. ECG also demonstrated prognosis significance in terms of future cardiovascular events in this population.

## Supplementary Information

**Additional file 1: Supplemental Table 1.** Specific ECG Patterns and Risk of Myocardial Ischemia. **Supplemental Table 2.** Study Outcomes Data.

## Data Availability

The datasets used and/or analyzed during the current study are available from the corresponding author on reasonable request.

## Abbreviations

CAD: Coronary artery disease
CI: Confidence interval
CMR: Cardiac magnetic resonance
ECG: Electrocardiography
EF: Ejection fraction
FOV: Field of view
HR: Hazard ratio
LGE: Late gadolinium enhancement
LV: Left ventricular
MACE: Major adverse cardiac events
MI: Myocardial infarction
SD: Standard deviation
SSFP: Steady-state free precession
TE: Echo time
TR: Repetitive time
