# Supplementary material for: Clinical values of resting electrocardiography in patients with known or suspected chronic coronary artery disease: a stress perfusion cardiac MRI study
Source: BMC Cardiovasc Disord. 2021 Dec 28;21:621. doi: 10.1186/s12872-021-02440-5 (PMC8714441; doi:10.1186/s12872-021-02440-5)
Supplement: Supplementary file 1 — Additional file 1: Supplemental Table 1. Specific ECG Patterns and Risk of Myocardial Ischemia. Supplemental Table 2. Study Outcomes Data. [file 12872_2021_2440_MOESM1_ESM.docx]

**Supplemental Materials**

**Supplemental Table 1.** Specific ECG Patterns and Risk of Myocardial Ischemia.

|  | Myocardial ischemia | |
| --- | --- | --- |
|  | Frequency | Percent (95%CI) |
| All patients (n=349) | 83 | 24 (19, 28) |
| No ECG abnormality (n=122) | 21 | 17 (10, 24) |
| Major ECG abnormality (n=106)  Q-Qs wave (n=34)  Left ventricular hypertrophy (n=25)  Complete bundle branch/IVB (n=26)  Atrial fibrillation/flutter (n=6)  Major ST-T changes (n=36) | 39  16  7  8  1  17 | 37 (27, 46)  47 (29, 65)  28 (9, 47)  31 (12, 50)  17 (-26, 60)  47 (30, 64) |
| Minor ECG abnormality (n=121)  Minor ST-T changes (n=68)  Minor/isolated Q wave (n=71) | 23  13  13 | 19 (12, 26)  19 (10, 29)  18 (9, 28) |

CI = confidence interval; ECG = electrocardiographic; IVB = intraventricular block.

**Supplemental Table 2.** Study Outcomes Data.

|  | All  (n=495) | Major ECG  Abnormality  (n=185) | Minor ECG  Abnormality  (n=154) | No ECG  Abnormality  (n=156) |
| --- | --- | --- | --- | --- |
| Major adverse cardiac events  Cardiac mortality  Nonfatal myocardial infarction  Hospitalization for unstable angina  Hospitalization for heart failure  Late revascularization | 91 (18.4)  13 (2.6)  29 (5.9)  7 (1.4)  52 (10.5)  28 (5.7) | 56 (30.3)  10 (5.4)  16 (8.6)  1 (0.5)  37 (20)  16 (8.6) | 14 (9.1)  1 (0.6)  8 (5.2)  2 (1.3)  5 (3.2)  8 (5.2) | 21 (13.5)  2 (1.3)  5 (3.2)  4 (2.6)  10 (6.4)  4 (2.6) |

Values are numbers (percentages).

ECG = electrocardiographic.
